# Time-restricted eating in overweight and obese adults: an evidence summary and clinical recommendations

**DOI:** 10.1186/s41043-025-01221-6

**Published:** 2026-01-13

**Authors:** Hui Liu, Zhuolian Zheng, Fuliang Shangguan, Yu Guo, Huixi Yu, Juping Yu, Yinhua Su, Zhongyu Li

**Affiliations:** 1 School of Nursing, University of South China, No. 28, Changsheng West Road, Hengyang, 421001 Hunan China; 2 Faculty of Life Sciences and Education, University of South Wales, Pontypridd, CF374BD Wales UK

**Keywords:** Time-restricted eating, overweight, Obesity, Diet, Intervention, Nutrition

## Abstract

**Objective:**

This systematic review aims to synthesize the current evidence and develop evidence-based recommendations regarding time-restricted eating (TRE) for weight management in adults with overweight and obesity, addressing a gap in specific clinical guidelines.

**Methods:**

We conducted a systematic search of nine databases and six websites for relevant literature up to September 2024. Included studies comprised randomized controlled trials (RCTs), clinical guidelines, expert consensus statements, and systematic reviews focusing on TRE in the target population. Two reviewers independently performed study selection, data extraction, and methodological quality assessment using standardized tools (e.g., AMSTAR 2, AGREE II, JBI checklists). Evidence was synthesized thematically, and recommendations were graded using the JBI framework.

**Results:**

The search identified 5535 records. After screening, 25 articles were included: five guidelines, three expert consensuses, eight systematic reviews, and nine RCTs. The synthesis yielded 39 key evidence points across six domains: applicable populations, intervention protocols, dietary considerations, psychological and sleep effects, efficacy, and safety. The synthesized evidence suggests that TRE can induce significant weight loss and improve cardiometabolic parameters (e.g., blood glucose and lipid profiles) in the short to medium term. While heterogeneity exists across individual studies, this review identifies key factors (e.g., eating window protocols, adherence) that may influence outcomes and provides a framework for clinical decision-making.

**Conclusions:**

TRE represents a promising dietary intervention for adults with overweight and obesity. This review provides a structured evidence summary and practical recommendations to guide its clinical application. Future research should focus on the long-term efficacy, sustainability, and impact of TRE on hard clinical endpoints.

**Level of evidence:**

Level I, systematic review.

**Supplementary Information:**

The online version contains supplementary material available at 10.1186/s41043-025-01221-6.

## Introduction

The global prevalence of overweight and obesity has reached epidemic proportions, presenting a formidable burden on healthcare systems worldwide [1–3]. Obesity is a major risk factor for numerous chronic diseases, including type 2 diabetes, cardiovascular diseases, and certain cancers, leading to increased morbidity and mortality [4–6]. While traditional dietary interventions, such as continuous calorie restriction, are effective for weight loss, their long-term success is often limited by poor adherence, metabolic adaptation, and psychological fatigue [7–9].

Time-restricted eating (TRE), a form of intermittent fasting, has emerged as a promising alternative strategy [10]. It focuses on consolidating daily caloric intake within a consistent window of time (typically 4 to 10 h), followed by a prolonged fasting period [11]. This approach may offer advantages by aligning food intake with circadian biology, potentially improving metabolic regulation and simplifying dietary adherence compared to traditional calorie-counting methods [12, 13]. A growing body of evidence, including several randomized controlled trials (RCTs) and meta-analyses, suggests that TRE can promote weight loss and improve cardiometabolic health markers, such as insulin sensitivity and blood lipid profiles, in populations with overweight and obesity [14–18].

However, the evidence base is not conclusive. Some well-designed RCTs have reported no significant advantage of TRE over standard dietary advice for weight loss or metabolic improvement [19, 20]. Furthermore, significant heterogeneity exists across studies regarding key intervention parameters, such as the duration and timing of the eating window, the degree of concurrent caloric restriction, and participant characteristics. These inconsistencies have resulted in a lack of clear, consolidated guidance for clinicians seeking to implement TRE in practice.

Although previous systematic reviews have synthesized the efficacy data of TRE, there remains a need for a comprehensive evidence summary that integrates not only RCT findings but also clinical guidelines and expert consensus statements. Such integration is crucial for translating research findings into practical, graded recommendations that address real-world clinical questions—such as patient selection, intervention protocols, and safety monitoring.

Therefore, this systematic review aims to address this gap by, firstly, synthesizing the highest levels of evidence (including guidelines, consensus statements, systematic reviews, and RCTs on TRE for overweight and obesity. Secondly, it will critically appraise this evidence to identify sources of heterogeneity. Finally, based on this synthesis, it will generate structured, evidence-based recommendations to guide clinical practice and future research.

## Methods

### Search strategy

This review was conducted according to an a priori protocol registered on the PROSPERO platform (CRD42024507590). The research question was formulated using the PIPOST framework [21], which defines the **P**opulation (adults with overweight/obesity), **I**ntervention (time-restricted eating, TRE), **P**rofessionals (healthcare providers), **O**utcomes (anthropometric, metabolic, psychosocial measures), **S**etting (clinical/community), and **T**ype of evidence (guidelines, consensus, systematic reviews, RCTs).

A comprehensive literature search was conducted from database inception up to September 2024. The following nine databases were searched: China National Knowledge Infrastructure (CNKI), Wanfang Data, VIP Database for Chinese Technical Periodicals (VIP), SinoMed, Cochrane Library, Embase, Joanna Briggs Institute (JBI), PubMed, and Web of Science. We also searched six relevant websites: UpToDate, Guidelines International Network (GIN), National Institute for Health and Care Excellence (NICE), World Health Organization (WHO), Agency for Healthcare Research and Quality (AHRQ), and American Medical Association (AMA).

A comprehensive literature search was conducted from database inception up to September 2024 across nine databases and six websites (listed in Supplementary Table S1). The search strategy employed a combination of controlled vocabulary and free-text terms. For example, the PubMed search strategy was: (((((((((((Intermittent Fasting[MeSH Terms]) OR (Intermittent Fasting[Text Word])) OR (Fasting, Intermittent[Title/Abstract])) OR (Time Restricted Eating[Title/Abstract])) OR (Eating, Time Restricte[Title/Abstract])) OR (Time Restricted Fasting[Title/Abstract])) OR (Fasting, Time Restricted[Title/Abstract])) OR (Restricted Fastings, Time[Title/Abstract])) OR (Time Restricted Feeding[Title/Abstract])) OR (Feeding, Time Restricted[Title/Abstract])) OR (Time Restricted Feedings[Title/Abstract])) AND (((Obesity[MeSH Terms]) OR (Obesity[Title/Abstract])) OR ((Overweight[MeSH Terms]) OR (Overweight[Title/Abstract]))). The complete search strategies for all sources are provided in Supplementary Table S1.

### Study selection

Inclusion criteria were: (1) participants aged 18 years or older with overweight or obesity; (2) intervention involving any form of TRE; (3) study design being a clinical guideline, expert consensus statement, systematic review, or RCT; (4) publication in English or Chinese.

Exclusion criteria were: (1) studies with an ambiguous or insufficient description of the TRE protocol; (2) studies lacking relevant objective outcome measures; (3) duplicate publications; (4) full text unavailable; (5) articles judged to be of low methodological quality based on our pre-defined quality assessment thresholds (see Sect. 2.4).

### Data extraction

All retrieved records were managed using NoteExpress software, and duplicates were removed. A standardized data extraction form was developed. Two reviewers (H.L. and Z.Z.) independently extracted data from the included articles. The extracted information included: publication year, authors, study design, detailed TRE intervention characteristics (e.g., eating window, duration), participant demographics, comparator details, outcome measures, and key findings. Any discrepancies in extraction were resolved through discussion or, if necessary, by consulting a third researcher (F.S.).

### Quality assessment

The methodological quality of included studies was independently assessed by two reviewers (H.L. and Z.Z.) using standardized critical appraisal tools. Disagreements were resolved by consensus or by arbitration from a third reviewer (F.S.).

**For RCTs**, the revised JBI Critical Appraisal Tool for Randomized Controlled Trials was used [22]. This tool assesses risk of bias across 13 domains.

**For expert consensus and opinion articles**, the JBI Critical Appraisal Checklist for Text and Opinion Papers was used [23].

**For clinical guidelines**, the Appraisal of Guidelines for Research and Evaluation II (AGREE II) instrument was used [24]. Guidelines scoring below 30% in more than three of the six domains (i.e., rated ‘C’) were excluded from the final synthesis.

**For systematic reviews**, the Assessment of Multiple Systematic Reviews 2 (AMSTAR 2) tool was used [25]. Reviews rated as ‘critically low’ quality (meeting ≤ 3 of the 16 criteria) were excluded.

### Evidence synthesis and classification

Two reviewers (H.L. and F.S.) independently performed a qualitative synthesis of the extracted evidence. Discrepancies in interpretation were resolved through team discussion. The evidence was organized into thematic domains. The level of evidence for each individual piece was classified from Level I (highest) to Level V (lowest) according to the JBI Evidence Pre-classification System (2014) [26]. When recommendations from different sources conflicted, precedence was given to evidence from higher-level study designs and more recent publications.

### Evidence recommendation

The strength of each recommendation was graded as either **A (strong recommendation)** or **B (weak recommendation)**. This grading was determined through team consensus based on the JBI FAME scale, which evaluates the Feasibility, Appropriateness, Meaningfulness, and Effectiveness of the evidence within the context of clinical practice [27].

## Results

### Search results

**Fig. 1:**
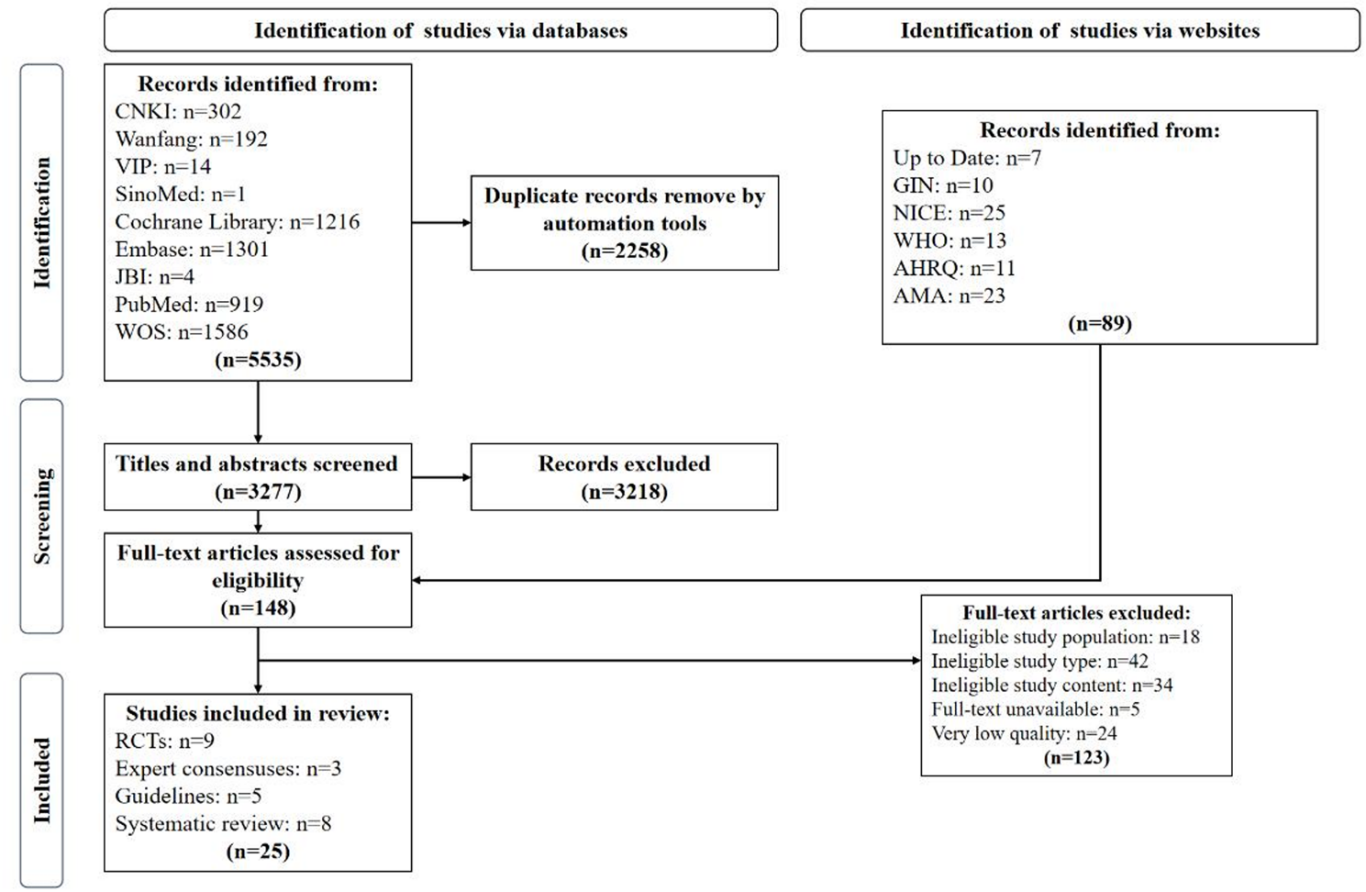
Flow diagram of literature search and screen

The systematic search across databases and websites yielded 5,535 records. After removing 2,258 duplicates, the titles and abstracts of 3,277 records were screened. Following this, 148 full-text articles were assessed for eligibility. Of these, 123 articles were excluded for the following reasons: 18 did not meet population criteria, 42 were of an ineligible study type, 34 did not meet the content requirements regarding TRE, 5 were unavailable in full text, and 24 were of low methodological quality. Ultimately, 25 articles met all inclusion criteria and were included in the final synthesis: five clinical guidelines, three expert consensus statements, eight systematic reviews, and nine RCTs. The study selection process is detailed in the PRISMA flow diagram (Fig. 1).

### Quality evaluation results

RCTs The nine included RCTs were conducted in the United States (*n* = 4), Brazil (*n* = 2), China (*n* = 1), Poland (*n* = 1), and Germany (*n* = 1) [19, 20, 28–34]. Quality assessment using the revised JBI tool indicated an overall low-to-moderate risk of bias. The most common limitations were the inherent difficulty of blinding participants to a dietary intervention and, in some cases, unclear allocation concealment. The detailed assessment for each RCT is presented in Supplementary Table S2.

Expert consensus All three included consensus statements were deemed acceptable in quality upon appraisal with the JBI checklist for text and opinion, as they clearly stated their aims, were based on appropriate expert experience, and referenced the extant literature [35–37]. No articles in this category were excluded based on quality (see Supplementary Table S3).

**Guidelines**

The five included guidelines were appraised using the AGREE II instrument [38–42]. The mean scores across the six domains were: scope and purpose (98.1%), stakeholder involvement (90.6%), rigor of development (89.2%), clarity of presentation (82.5%), applicability (67.9%), and editorial independence (100%). All guidelines were rated ‘A’ (recommended for use) or ‘B’ (recommended with modifications) and were thus retained (Supplementary Table S4).

Systematic reviews The eight included systematic reviews, encompassing 100 primary studies, were evaluated with the AMSTAR 2 tool [43–50]. Six were rated as high quality and two as moderate quality; none were rated critically low. Therefore, all eight were included in the evidence synthesis (Supplementary Table S5).

### Evidence synthesis

Thematic synthesis of the 25 included articles generated 39 discrete pieces of evidence. For clarity and to highlight the main clinical messages, these were organized into six overarching domains: (1) Applicable and Contraindicated Populations, (2) Intervention Protocol (Time and Procedure), (3) Diet and Nutritional Considerations, (4) Psychological and Sleep Impacts, (5) Intervention Efficacy, and (6) Safety and Risk Management. Table 1 presents a concise, integrated summary of the key recommendations within these domains. The complete and detailed evidence table, listing all 39 items with their source, level of evidence, and recommendation grade, is available as Supplementary Table S6.

**Table 1:** Summary of evidence and recommendations for TRE in adults with overweight/obesity

Domain	Key Evidence/Recommendation	Level of Evidence	Strength of Recommendation
**1. Applicability**	TRE is suitable for most adults with overweight/obesity. It is contraindicated in pregnancy, lactation, eating disorders, and severe/uncontrolled metabolic diseases.	II	A
**2. Intervention Protocol**	An 8-hour eating window (e.g., 8:00–16:00) is commonly effective. Windows < 6 h may increase adverse events; >10 h may diminish efficacy. Effects are typically seen after 4 weeks, significant by 12 weeks.	I	A
**3. Diet & Nutrition**	Diet quality must be emphasized. Caloric restriction combined with TRE may enhance weight loss. Adequate hydration and fiber intake are recommended.	I/II	A
**4. Psychosocial & Sleep**	TRE may improve eating behaviors. Initial sleep disruption or anxiety in susceptible individuals is possible; psychological support is beneficial.	I/II	A
**5. Efficacy**	TRE is effective for short-term weight loss and improves cardiometabolic parameters (glucose, lipids, blood pressure).	I	A
**6. Safety**	TRE is generally safe. Common side effects (hunger, headache) are mild and transient. Risk is minimized with proper screening and monitoring.	I	A

## Discussion

### Summary of main findings and clinical integration

This systematic review synthesizes evidence from 25 diverse sources to create a structured, evidence-based framework for implementing TRE in adults with overweight and obesity. The 39 consolidated pieces of evidence and the resulting recommendations address critical clinical questions beyond simple efficacy, encompassing patient selection, intervention tailoring, safety, and support mechanisms. Notably, by integrating evidence across different study types (guidelines, consensus, reviews, RCTs) and geographical sources, this work provides a more comprehensive and balanced perspective than reviews focusing solely on RCTs. Our findings indicate that TRE is a feasible and effective short-to-medium-term strategy for weight loss and cardiometabolic improvement for most, but not all, individuals in this population. The synthesis clarifies that successful application depends significantly on careful protocol design and individualized patient management.

### Interpretation of key evidence and heterogeneity

Consistent with several prior meta-analyses, this review confirms that TRE can induce clinically meaningful weight loss (3 ~ 5%) and improve key metabolic parameters such as fasting glucose and lipid profiles [51, 52]. The proposed mechanisms, including alignment with circadian rhythms and extended nightly fasting, provide a physiological rationale for these benefits. However, our synthesis also explicitly highlights the heterogeneity in outcomes reported across individual RCTs, a finding underscored by the conflicting results of high-quality studies. This variability can largely be attributed to differences in critical intervention parameters—such as the timing and duration of the eating window, the presence or absence of concurrent caloric advice, and baseline participant characteristics—which our evidence summary helps to delineate. This underlines the importance of moving beyond a one-size-fits-all approach and supports the need for the personalized implementation framework this review provides.

### Clinical implications and practical application

The synthesized evidence translates into clear practical steps for clinicians. First, patient selection is paramount; TRE is contraindicated for specific groups, and screening is essential. Second, the intervention protocol should be co-created with the patient. While an 8-hour window is a common and effective starting point, flexibility to fit individual lifestyles is key to long-term adherence. Third, nutritional quality must not be neglected; emphasizing whole foods, adequate protein, and hydration within the eating window is crucial for health and sustainability. Finally, ongoing support and monitoring from a multidisciplinary team, utilizing both in-person and digital tools, are vital components for success and safety. TRE should thus be viewed not merely as a dietary restriction but as a structured behavioral intervention supported within a clinical care framework.

### Limitations and future research directions

Several limitations must be considered when interpreting our findings. First, the heterogeneity among primary studies is addressed through our transparent evidence grading system, which allows users to gauge the certainty behind each recommendation. Second, while the inclusion of guidelines from diverse regions captures a wide range of perspectives, it also necessitates local adaptation of the recommendations, a principle emphasized in our framework. The most pressing limitation is the scarcity of long-term (> 12 months) RCT data, which restricts conclusions about sustainability and hard clinical endpoints. Future research should therefore prioritize long-term, pragmatic RCTs across diverse populations and the development of tools to predict individual response to TRE.

## Conclusion

This systematic review synthesizes and evaluates the current best available evidence on time-restricted eating (TRE) for weight management in adults with overweight and obesity. By integrating findings from clinical guidelines, expert consensuses, systematic reviews, and randomized trials, it provides a structured, evidence-based framework comprising 39 key pieces of evidence and practical recommendations. The evidence supports TRE as a viable and generally safe intervention that can promote weight loss and improve cardiometabolic health in the short to medium term, provided it is implemented with careful attention to patient selection, individualized protocol design, and ongoing support. This work clarifies the often heterogeneous literature, offering clinicians a nuanced guide for practice and highlighting critical gaps—particularly the need for long-term outcome data—to direct future research.

## Supplementary Information

Supplementary Material 1

Supplementary Material 2

Supplementary Material 3

Supplementary Material 4

## Data Availability

All data generated or analysed during this study are included in this published article [and its supplementary information files].

## Abbreviations

TRE: Time-Restricted Eating
RCT: Randomized Controlled Trial
JBI: Joanna Briggs Institute
AGREE II: Appraisal of Guidelines for Research and Evaluation II
PRISMA: Preferred Reporting Items for Systematic Reviews and Meta-Analyses
AMSTAR 2: A Measurement Tool to Assess systematic Reviews 2
PROSPERO: International Prospective Register of Systematic Reviews

## Declarations

### PROSPERO registration number

CRD42024507590.
