# Supplementary material for: Time-restricted eating in overweight and obese adults: an evidence summary and clinical recommendations
Source: J Health Popul Nutr. 2026 Jan 13;45:53. doi: 10.1186/s41043-025-01221-6 (PMC12888743; doi:10.1186/s41043-025-01221-6)
Supplement: Supplementary file 2 — Supplementary Material 2 [file 41043_2025_1221_MOESM2_ESM.docx]

**S1****. Search strategy for databases and websites**

| **Date base/ website name** | **Search** |
| --- | --- |
| CNKI | (SU%='Intermittent Fasting' or SU%='Time Restricted Eating' or SU%='Time Restricted Feeding' or TKA%='Intermittent Fasting' or TKA%='Time Restricted Eating' or TKA%='Time Restricted Feeding') and (SU%='Overweight' or SU%='Obesity' or TKA%='Overweight' or TKA%='Obesity') |
| Wanfang | (Subject: (Intermittent Fasting OR Time Restricted Eating OR Time Restricted Feeding) OR Title/Keyword: (Intermittent Fasting OR Time Restricted Eating OR Time Restricted Feeding)) AND (Subject: (Overweight OR Obesity) OR Title/Keyword: (Overweight OR Obesity)) |
| VIP | (M=(Intermittent Fasting OR Time Restricted Eating OR Time Restricted Feeding) AND M=(Overweight OR Obesity)) |
| SinoMed | ("Intermittent Fasting"[Common Field: Intelligent] OR "Time Restricted Eating"[Common Field: Intelligent] OR "Time Restricted Feeding"[Common Field: Intelligent]) AND "Overweight OR Obesity"[Common Field: Intelligent] |
| Cochrane Library | (Intermittent Fasting OR Fasting, Intermittent OR Time Restricted Eating OR Eating, Time Restricte OR Time Restricted Fasting OR Fasting, Time Restricted OR Restricted Fastings, Time OR Time Restricted Feeding OR Feeding, Time Restricted OR Time Restricted Feedings) AND (Obesity OR Overweight) |
| Embase | ('intermittent fasting'/exp OR 'intermittent fasting' OR 'fasting, intermittent' OR 'time restricted eating'/exp OR 'time restricted eating' OR 'eating, time restricted' OR 'time restricted fasting' OR 'fasting, time restricted' OR 'restricted fastings, time' OR 'time restricted feeding'/exp OR 'time restricted feeding' OR 'feeding, time restricted' OR 'time restricted feedings') AND ('obesity'/exp OR 'obesity' OR 'overweight'/exp OR 'overweight') AND [<1966-2024]/py |
| **Date base/ website name** | **Search** |
| JBI | ((Intermittent Fasting).sh. or (Intermittent Fasting OR Fasting, Intermittent OR Time Restricted Eating OR Eating, Time Restricte OR Time Restricted Fasting OR Fasting, Time Restricted OR Restricted Fastings, Time OR Time Restricted Feeding OR Feeding, Time Restricted OR Time Restricted Feedings).ab, kw, ti.) and (Obesity OR Overweight).ab, kw, ti. |
| PubMed | (((((((((((Intermittent Fasting[MeSH Terms]) OR (Intermittent Fasting[Text Word])) OR (Fasting, Intermittent[Title/Abstract])) OR (Time Restricted Eating[Title/Abstract])) OR (Eating, Time Restricte[Title/Abstract])) OR (Time Restricted Fasting[Title/Abstract])) OR (Fasting, Time Restricted[Title/Abstract])) OR (Restricted Fastings, Time[Title/Abstract])) OR (Time Restricted Feeding[Title/Abstract])) OR (Feeding, Time Restricted[Title/Abstract])) OR (Time Restricted Feedings[Title/Abstract])) AND (((Obesity[MeSH Terms]) OR (Obesity[Title/Abstract])) OR ((Overweight[MeSH Terms]) OR (Overweight[Title/Abstract]))) |
| Web of Science | (TS=(Intermittent Fasting OR "Fasting, Intermittent" OR "Time Restricted Eating" OR "Eating, Time Restricted" OR "Time Restricted Fasting" OR "Fasting, Time Restricted" OR "Restricted Fastings, Time" OR "Time Restricted Feeding" OR "Feeding, Time Restricted" OR "Time Restricted Feedings") OR AB=(Intermittent Fasting OR "Fasting, Intermittent" OR "Time Restricted Eating" OR "Eating, Time Restricted" OR "Time Restricted Fasting" OR "Fasting, Time Restricted" OR "Restricted Fastings, Time" OR "Time Restricted Feeding" OR "Feeding, Time Restricted" OR "Time Restricted Feedings")) AND (TS=(Obesity OR Overweight) OR AB=(Obesity OR Overweight))  Date rage: all years (1637-2024) |
| Up to Date | Obesity  Overweight |
| **Date base/ website name** | **Search** |
| Guidelines International Network (GIN) | https://g-i-n.net/international-guidelines-library  Obesity  Overweight |
| National Institute for Healthcare and Excellence (NICE) | https://www.nice.org.uk/guidance  Obesity  Overweight |
| World Health Organization (WHO) | https://www.who.int/publications/who-guidelines  Obesity  Overweight |
| [Agency for Healthcare Research and Quality (AHRQ)](https://www.ahrq.gov/gam/index.html) | <https://www.ahrq.gov/gam/index.html>  obesity guidelines |
| American Medical Association(AMA) | <https://www.ama-assn.org/>  Obesity |
