# Supplementary material for: Time-restricted eating in overweight and obese adults: an evidence summary and clinical recommendations
Source: J Health Popul Nutr. 2026 Jan 13;45:53. doi: 10.1186/s41043-025-01221-6 (PMC12888743; doi:10.1186/s41043-025-01221-6)
Supplement: Supplementary file 3 — Supplementary Material 3 [file 41043_2025_1221_MOESM3_ESM.docx]

****Supplementary Table S6. Complete evidence table for time-restricted eating (TRE) in adults with overweight or obesity (n=39 items).****

| Item No. | Thematic Domain | Evidence Statement / Recommendation | Source(s) | Level of Evidence | Grade of Recommendation |
| --- | --- | --- | --- | --- | --- |
| **E1** | Applicability | Overweight defined as: WHO: 25 ≤ BMI < 30 kg/m²; Asian/African criteria: 23 ≤ BMI < 27.5 kg/m². | [41] | II b | A |
| **E2** | Applicability | Obesity defined as: WHO: BMI ≥ 30 kg/m²; Asian/African criteria: BMI ≥ 27.5 kg/m². | [41] | II b | A |
| **E3** | Applicability | Central obesity defined as: WHO: Men ≥90 cm, Women ≥80 cm; China: Men ≥90 cm, Women ≥85 cm. | [36] | V b | A |
| **E4** | Applicability | **Contraindication:** TRE is not recommended for individuals with nutritional disorders, perimenopause/irregular menstruation, cardiovascular disease, or those who are pregnant/lactating/trying to conceive. | [19, 29, 34] | I c | A |
| **E5** | Intervention Protocol | Eating windows longer than 10 hours may hinder weight loss, while windows shorter than 6 hours may increase adverse effects. | [19, 43, 50] | I a | A |
| **E6** | Intervention Protocol | An 8-hour eating window (e.g., 8:00-16:00) is a potentially effective strategy for weight management. | [44, 46] | I a | A |
| **E7** | Intervention Protocol | Effects of TRE typically emerge after 4 weeks, become significant within 12 weeks, and optimal results may be achieved within 6 months. | [37, 45, 43] | I a | B |
| ****E8**** | Intervention Protocol | For obesity with prediabetes, a weight loss target of 5-7% is recommended. | [35, 42] | II b | A |
| ****E9**** | Intervention Protocol | For obesity with diabetes, a weight loss target of 7-15% is recommended. | [37, 42] | II b | A |
| ****E10**** | Intervention Protocol | For obesity with other comorbidities, a weight loss target of 10% is recommended. | [41] | II b | A |
| ****E11**** | Intervention Protocol | Effective counseling methods (e.g., motivational interviewing) help promote desired behavioral change. | [41] | II b | A |
| ****E12**** | Intervention Protocol | Various educational methods (e.g., food diaries, nutrition apps) under professional guidance can improve cognitive skills and eating control. | [36] | V b | B |
| ****E13**** | Intervention Protocol | A multidisciplinary team (doctors, nurses, dietitians) should be involved in weight management. | [36, 38] | II b | A |
| ****E14**** | Intervention Protocol | Support from family, friends, or partners is beneficial. | [39-40] | II b | A |
| ****E15**** | Intervention Protocol | Interventions can be offered in various community settings (e.g., community centers, workplaces). | [41] | II b | A |
| ****E16**** | Intervention Protocol | Face-to-face guidance (one-on-one or group sessions) is effective. | [40, 41] | II b | A |
| ****E17**** | Intervention Protocol | Remote guidance (telephone, video, electronic records) is feasible and effective. | [20, 31, 41] | I c | A |
| ****E18**** | Intervention Protocol | Regular contact (e.g., every 3 months) and more frequent initial meetings (weekly/biweekly) support behavior change. | [41] | II b | A |
| ****E19**** | Diet & Nutrition | Discuss dietary options with patients. | [35, 38-42] | II b | A |
| ****E20**** | Diet & Nutrition | Dietary planning should be based on personal preferences, health status, social environment, and previous treatment. | [34, 40, 41] | I c | A |
| ****E21**** | Diet & Nutrition | Combining TRE with caloric restriction can enhance weight loss effects. | [44, 45] | I a | A |
| ****E22**** | Diet & Nutrition | For weight loss, energy intake can be set at 85% (overweight) or 80% (obesity) of normal requirements, or reduced by 30-50%/500 kcal daily. | [36] | V b | B |
| ****E23**** | Diet & Nutrition | Ensure adequate hydration (~2 L/day), consider pre/probiotics, and maintain regular bowel movements to reduce adverse events. | [43] | I a | A |
| ****E24**** | Psychosocial & Sleep | Overweight/obesity or weight loss failure can cause psychological distress, potentially inducing anxiety or depression. | [36] | V b | A |
| ****E25**** | Psychosocial & Sleep | Provide psychological counseling and support. | [36] | V b | A |
| ****E26**** | Psychosocial & Sleep | Implement educational and regulatory measures to prevent weight stigma and discrimination. | [37] | V b | A |
| ****E27**** | Psychosocial & Sleep | Encourage patients and obtain consent before discussing obesity-related issues. | [40] | II b | A |
| ****E28**** | Psychosocial & Sleep | Initial sleep disturbances (e.g., difficulty falling asleep) may occur; sleep quality can be assessed via the Pittsburgh Sleep Quality Index. | [20] | I c | A |
| ****E29**** | Psychosocial & Sleep | Improving sleep and stress-coping styles is beneficial. | [39] | II b | A |
| ****E30**** | Psychosocial & Sleep | Follow-up contact should be provided at least every 3 months after intervention completion. | [41] | II b | A |
| ****E31**** | Psychosocial & Sleep | Follow-up can be conducted face-to-face or remotely. | [41] | II b | A |
| ****E32**** | Psychosocial & Sleep | Follow-up should monitor adherence (days of TRE adherence) and weight maintenance. | [20, 33] | I c | A |
| ****E33**** | Efficacy | TRE reduces body weight and BMI. | [20, 30, 34, 43, 44, 49] | I a | A |
| ****E34**** | Efficacy | TRE reduces fasting blood glucose and insulin resistance, improving metabolism. | [31, 50] | I a | A |
| ****E35**** | Efficacy | TRE reduces cardiovascular risk factors (e.g., lipids, blood pressure). | [29, 43, 44] | I a | A |
| ****E36**** | Efficacy | TRE helps reduce appetite levels and improve eating habits. | [28, 33] | I c | A |
| ****E37**** | Safety | Short eating windows may cause adverse reactions (e.g., dizziness, hypoglycemia, gastrointestinal discomfort). | [43] | I c | A |
| ****E38**** | Safety | TRE may pose risks of hunger, decreased sleep quality, and unbalanced nutrient intake. | [43] | I c | A |
| ****E39**** | Safety | Patients can adhere well to the TRE program with proper guidance and supervision. | [20, 28, 30, 31, 33, 34] | I c | A |
