# Supplementary material for: Time-restricted eating in overweight and obese adults: an evidence summary and clinical recommendations
Source: J Health Popul Nutr. 2026 Jan 13;45:53. doi: 10.1186/s41043-025-01221-6 (PMC12888743; doi:10.1186/s41043-025-01221-6)
Supplement: Supplementary file 4 — Supplementary Material 4 [file 41043_2025_1221_MOESM4_ESM.docx]

| **Table S2.** The quality assessment of the RCTs | | | | | | | | | | | | | | |
| --- | --- | --- | --- | --- | --- | --- | --- | --- | --- | --- | --- | --- | --- | --- |
| **Number** | **Reference** | **Item 1** | **Item 2** | **Item 3** | **Item 4** | **Item 5** | **Item 6** | **Item 7** | **Item 8** | **Item 9** | **Item 10** | **Item 11** | **Item 12** | **Item 13** |
| 1 | [19] | Yes | Unclear | Yes | Unclear | Unclear | Yes | Unclear | Yes | Yes | Yes | Yes | Yes | Yes |
| 2 | [20] | Yes | Yes | Yes | Unclear | Unclear | Yes | Unclear | Yes | Yes | Yes | Yes | Yes | Yes |
| 3 | [28] | Yes | Yes | Yes | Unclear | Unclear | Yes | Yes | Yes | Yes | Yes | Yes | Yes | Yes |
| 4 | [29] | Yes | Yes | Yes | Unclear | Unclear | Yes | Yes | Yes | Yes | Yes | Yes | Yes | Yes |
| 5 | [30] | Yes | Unclear | Yes | Unclear | Unclear | Yes | NO | Yes | Yes | Yes | Yes | Yes | Yes |
| 6 | [31] | Yes | Yes | Yes | Yes | Yes | Yes | Yes | Yes | Yes | Yes | Yes | Yes | Yes |
| 7 | [32] | Yes | Unclear | Yes | Unclear | Unclear | Yes | Yes | Yes | Yes | Yes | Yes | Yes | Yes |
| 8 | [33] | Yes | Unclear | Yes | Unclear | NO | Yes | NO | Yes | Yes | Yes | Unclear | Yes | Yes |
| 9 | [34] | Yes | Unclear | Yes | Unclear | Unclear | Yes | Unclear | Yes | Yes | Yes | Yes | Yes | Yes |

| **Table S3.** The quality assessment of the expert consensuses | | | | | | | | |
| --- | --- | --- | --- | --- | --- | --- | --- | --- |
| **Number** | **Reference** | **Item 1** | **Item 2** | **Item 3** | **Item 4** | **Item 5** | **Item 6** | **Overall appraisal** |
| 1 | [35] | Yes | Yes | Yes | Yes | Yes | Not applicable | Include |
| 2 | [36] | Yes | Yes | Yes | Yes | Yes | Not applicable | Include |
| 3 | [37] | Yes | Yes | Yes | Yes | Yes | Not applicable | Include |

| **Table S4.** The characteristics of the guidelines and the scores for each domain | | | | | | | | |
| --- | --- | --- | --- | --- | --- | --- | --- | --- |
| **Number** | **Reference** | **(1) Scope and purpose (%)** | **(2) Stakeholder involvement (%)** | **(3) Rigor of development (%)** | **(4) Clarity of presentation (%)** | **(5) Applicability (%)** | **(6) Editorial independence (%)** | **Overall quality** |
| 1 | [38] | 98.61 | 94.44 | 92.71 | 90.28 | 76.04 | 100 | A |
| 2 | [39] | 98.61 | 93.06 | 89.58 | 86.11 | 57.29 | 100 | B |
| 3 | [40] | 100 | 98.61 | 96.35 | 98.61 | 86.46 | 100 | A |
| 4 | [41] | 97.22 | 87.5 | 87.5 | 76.39 | 67.71 | 100 | A |
| 5 | [42] | 95.83 | 79.17 | 80.21 | 61.11 | 52.08 | 100 | B |
| **Average score** | | 98.05 | 90.56 | 89.27 | 82.5 | 67.92 | 100 |  |

| **Table S5.** The quality assessment of the systematic reviews | | | | | | | | | | | | | | | | | | | |
| --- | --- | --- | --- | --- | --- | --- | --- | --- | --- | --- | --- | --- | --- | --- | --- | --- | --- | --- | --- |
| **Number** | **Reference** | **Items** | | | | | | | | | | | | | | | | **Quality level** | **Overall appraisal** |
|  |  | **1** | **2** | **3** | **4** | **5** | **6** | **7** | **8** | **9** | **10** | **11** | **12** | **13** | **14** | **15** | **16** |  |  |
| 1 | [43] | Yes | Partial Yes | Yes | Yes | Yes | Yes | Yes | Yes | Yes | No | Partial Yes | Yes | Yes | Yes | Partial Yes | Yes | High | Include |
| 2 | [44] | Yes | Yes | Yes | Yes | Yes | Yes | Yes | Yes | Yes | No | Yes | Yes | Yes | Yes | Yes | Yes | High | Include |
| 3 | [45] | Yes | Yes | Yes | Yes | Yes | Yes | Yes | Yes | Yes | No | Yes | Yes | Yes | Yes | Yes | Yes | High | Include |
| 4 | [46] | Yes | Partial Yes | Yes | Yes | Yes | Yes | Partial Yes | Yes | Yes | No | Yes | Yes | Yes | Yes | Yes | Yes | High | Include |
| 5 | [47] | Partial Yes | Partial Yes | Yes | Yes | Yes | Yes | Partial Yes | Yes | No | No | Yes | No | No | Yes | No | Yes | Medium | Include |
| 6 | [48] | Yes | Yes | Yes | Yes | Yes | Yes | Partial Yes | Yes | Yes | No | Partial Yes | Yes | Yes | Yes | Partial Yes | Yes | High | Include |
| 7 | [49] | Yes | No | Yes | Yes | Yes | Yes | Partial Yes | Yes | Yes | No | Yes | Yes | Yes | Yes | Yes | Partial Yes | Medium | Include |
| 8 | [50] | Yes | Yes | Yes | Yes | Yes | Yes | No | Yes | Yes | No | Yes | Yes | Yes | Yes | Yes | Yes | High | Include |
